# A Modal Rendition of ENSO Diversity

**DOI:** 10.1038/s41598-019-50409-4

**Published:** 2019-09-30

**Authors:** Rajib Chattopadhyay, Shivsai Ajit Dixit, B. N. Goswami

**Affiliations:** 1 Indian Institute of Tropical Meteorology, Pune, India; 2 Cotton University, Guwahati, India

**Keywords:** Atmospheric dynamics, Ocean sciences, Physical oceanography

## Abstract

The El Nino and Southern Oscillation (ENSO) ‘diversity’ has been considered as a major factor limiting its predictability, a critical need for disaster mitigation associated with the trademark climatic swings of the ENSO. Improving climate models for ENSO forecasts relies on deeper understanding of the ENSO diversity but currently at a nascent stage. Here, we show that the ENSO diversity thought previously as ‘complex,’ arises largely as varied contributions from three leading modes of the ENSO to a given event. The ENSO ‘slow manifold’ can be fully described by three leading predictable modes, a quasi-quadrennial mode (QQD), a quasi-biennial (QB) mode and a decadal modulation of the quasi-biennial (DQB). The modal description of ENSO provides a framework for understanding the predictability of and global teleconnections with the ENSO. We further demonstrate it to be a useful framework for understanding biases of climate models in simulating and predicting the ENSO. Therefore, skillful prediction of all shades of ENSO depends critically on the coupled models’ ability to simulate the three modes with fidelity, providing basis for optimism for future of ENSO forecasts.

## Introduction

The largest signal of climate variability on inter-annual time scale arising from ocean-atmosphere interaction in the tropics^1,2^, the El Nino and Southern Oscillation (ENSO) has deep and wide ranging impacts across the globe^3,4^. Early warning of amplitude and evolutionary history of an impending ENSO event, therefore, is critical for disaster mitigation and planning for adaptation strategy. As a consequence, ENSO prediction has remained on the top of agenda of the climate prediction community. Since the first failed attempt to predict the 1975 El Nino^5^ and the first successful forecast^6^, the ENSO predictions have made great strides^7,8^. However, the skill of even the latest models falls far short of the limit of potential predictability^9–11^. More importantly, the models fail to predict the observed event-to-event variability in evolution and amplitude of ENSO as highlighted by the failure of almost all models in predicting the 2014–15 event when almost all models predicted an ‘monster’ El Nino that failed to appear^12–14^. The event-to-event variability of the ENSO is now recognized to be a manifestation of the ‘diversity’ of the ENSO^15,16^. Recognition that ENSO occurs in various ‘flavours’ or ‘types’ has replaced the earlier thinking of a ‘canonical’ El Nino^17^. In particular, two prominent types, namely an eastern Pacific (EP) type El Nino and a Central Pacific (CP) type El Nino have been identified^18–20^. Therefore, it is not surprising that a great deal of current research on ENSO is focused around space-time characteristics, predictability of the ENSO diversity and ability of climate models to simulate it^15,16,21–23^. Despite identification of the two types of El Ninos, the ‘complexity’ of the ENSO^24^ with each event tending to have different spatial characteristics, temporal evolution and influence of the high frequency westerly winds bursts or impact of extra-tropical dynamics on them, continues to remain a road block for improving prediction skill of the models.

In view of the immense importance of clearly understanding the air-sea interactions associated with it, its teleconnections, predictability and impact around the globe, the understanding of ENSO diversity is critical, in particular in regard to the categorization of the two types, namely the CP and EP El Ninos. The motivation and objective of our study is based on our conviction that progress in prediction and predictability of ENSO could be achieved if we could describe the complex diversity of ENSO in terms of few ‘simpler’ units, namely leading modes of the coupled system. All studies, so far, relate the first two empirical orthogonal functions (EOFs) of sea surface temperature (SST) over the Pacific to the EP and CP El Ninos respectively^16,19,24^ that have been assumed as two different ‘modes’ of ENSO. Unfortunately, the first two EOFs are unlikely to be independent physical ‘modes’. In a conventional EOF analysis, while the spatial patterns are orthogonal by construction, the temporal characteristics (periodicity and temporal evolution of the spatial pattern) are not constrained to be independent. As a result, both the principal components (PCs) associated with the first two EOFs contain considerable contributions from higher frequencies. For them to be considered as physical modes, in addition to the spatial patterns, the temporal as well as the evolutionary characteristics need to be distinct. In temporal domain, the ENSO is known to have contributions from a quasi-quadrennial, a quasi-biennial and a decadal/inter-decadal^25–28^ time scales. In order to arrive at a ‘modal’ description of the ENSO, we approach the problem from the opposite direction. We try to separate the temporal modes with different spatial patterns associated with them for which the evolutionary history would be distinct. This may be achieved using an Extended EOF (EEOF)^28^ analysis with a suitable number of lagged copies of the fields. Based on monthly data of SST and sea level pressure (SLP) over the tropical Pacific between 1854 and 2004, we carry out an EEOF analysis with lagged copies of the fields up to 18 months. This analysis reveals three distinct ENSO ‘modes’, a quasi-quadrennial mode, a quasi-biennial mode and a decadal modulation of the quasi-biennial mode with distinct spatial patterns as well as temporal evolution of the spatial patterns. We consider them as physical modes as they are distinct both in temporal as well as spatial characteristics. We unravel that the modes could be considered as three building blocks for the diversity and the complexity of the ENSO that are realized simply from varying contributions of the three modes to a particular event. Hence, in this paper we present the teleconnections, the predictability and air-sea interaction associated with the modes. We also show that the description of ENSO in terms of these three modes is a useful framework to diagnose and understand the biases of coupled models in simulating and predicting the ENSO.

Our study is distinct from two other studies that come close. The extended MEI index^25^ is a coupled index of ENSO derived from an EOF analysis of SST and SLP data. In such a case, the first three EOFs will lack full distinction due to lack of time-embedding information. In another important study, Yeo and Kim^29^ use only SST data but use time embedding information in the context of CSEOF. However, their focus was to include the global warming mode that emerges as dominant mode in their study. Our focus in this study has been to describe the ‘diversity’ of the natural ENSO mode. As a result, the identified modes are from de-trended data and represent the leading modes of natural ENSO variability.

## Results

### The three modes

**Figure 1:**
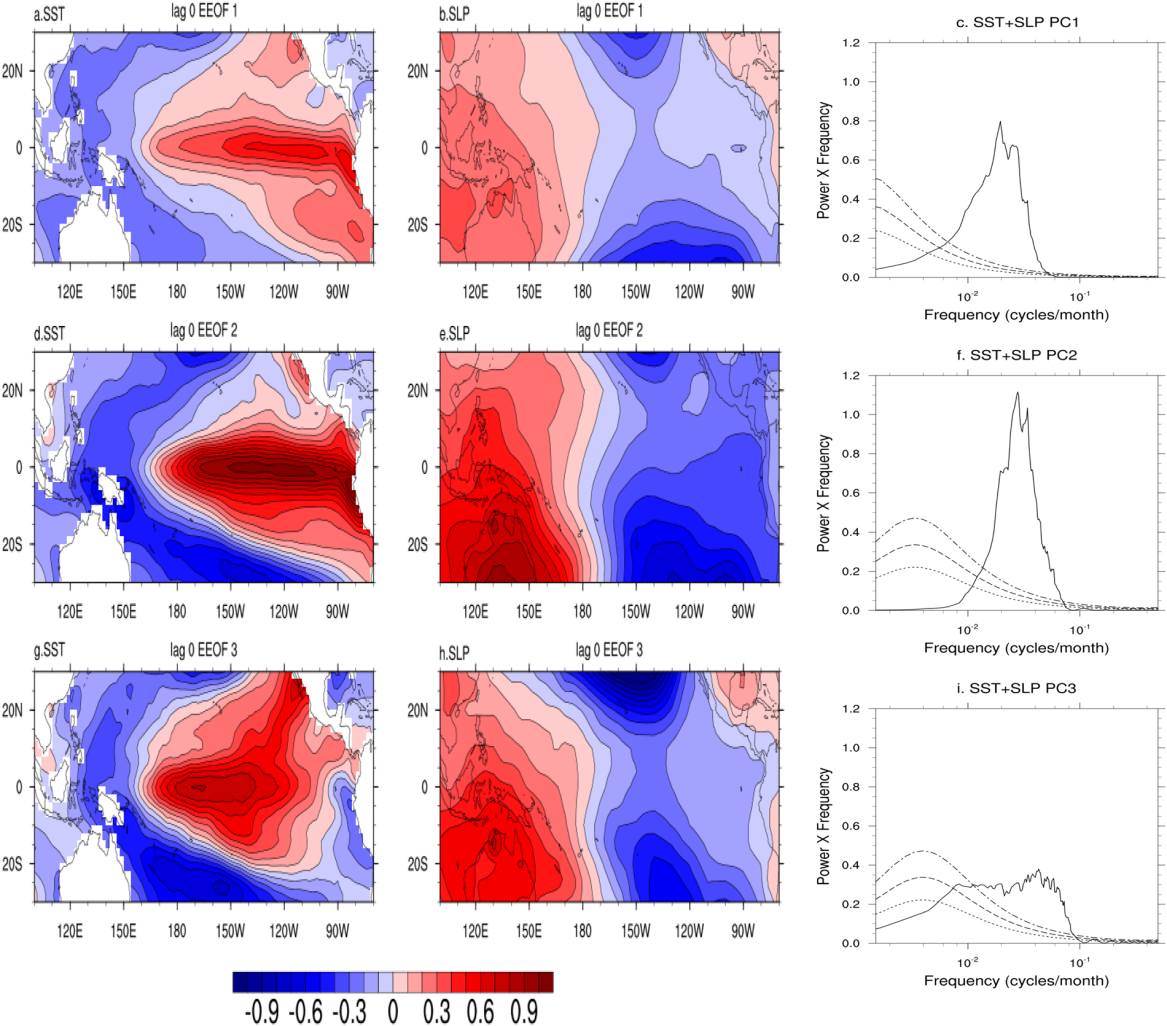
Plot of combined EEOF of SST and SLP based on HADSLP data and ERSST data for the Pacific (30°S–30°N; 100°E–90°W) and with time lag of 18 months used to create the co-variance matrix (see text). (**a**) EEOF-1 pattern for SST at lag 0, (**b**) EEOF-1 pattern of SLP at lag 0, (**c**) Power spectra of principal component of mode 1:PC1; (**d**–**f**) same as (**a**–**c**) but for mode 2 (EEOF2/PC2); (**g**–**i**) same as (**a**–**c**) but for mode 3 (EEOF3/PC3).

The first three EEOFs (Fig. 1) are considered as the ENSO modes as the dominant periodicity of all other EEOFs is closer to one year or less. The first EEOF represents a quasi-quadrennial (QQ) mode (Fig. 1c), the second EEOF a quasi-biennial (QB) mode (Fig. 1f) while the third EEOF representing a decadal modulation of the QB (DQB) mode (Fig. 1i). The decadal modulation of the QB mode is clearly evident in the time series of the PC3 (Fig. S1). Although the spatial patterns of the QQ mode and that of the QB mode have strong resemblance to that of the EP El Nino, the temporal evolutions of the spatial pattern are quite distinct (Fig. S2). While only the amplitude of the spatially stationary pattern of the QQ mode evolves in time (Fig. S2a), the spatial pattern of the QB mode starts at the coastal eastern Pacific and expands westward during the evolution (Fig. S2b). Although it has been known that some EL Ninos do evolve simultaneously over most of equatorial Pacific basin, while some others starts evolving from the east, here, we show that they indeed are two different modes of ENSO. Our analysis reveals that the EP El Ninos themselves have two different shades with very different evolutionary characteristics. On the other hand, the spatial structure of the DQB is similar to that of the CP El Nino. Not recognized so far, our analysis also brings out the unique character of the CP El Nino having a quasi-biennial character with SST anomalies starting from the equatorial coastal east Pacific and expanding to the central Pacific (Fig. S2c), bearing similarity to the QB mode. In addition to the mode having a decadal modulation, the central Pacific SST anomalies associated with the CP El Nino appear to be an extension of sub-tropical east Pacific SSTs, indicating an extra-tropical linkage for this mode (Fig. S2c). While the existence of a tropical Pacific decadal mode (TPDM) and its connection with extra-tropics has been known^27,28,30^, our analysis help formalize its association with the ENSO.

### Teleconnections

**Figure 2:**
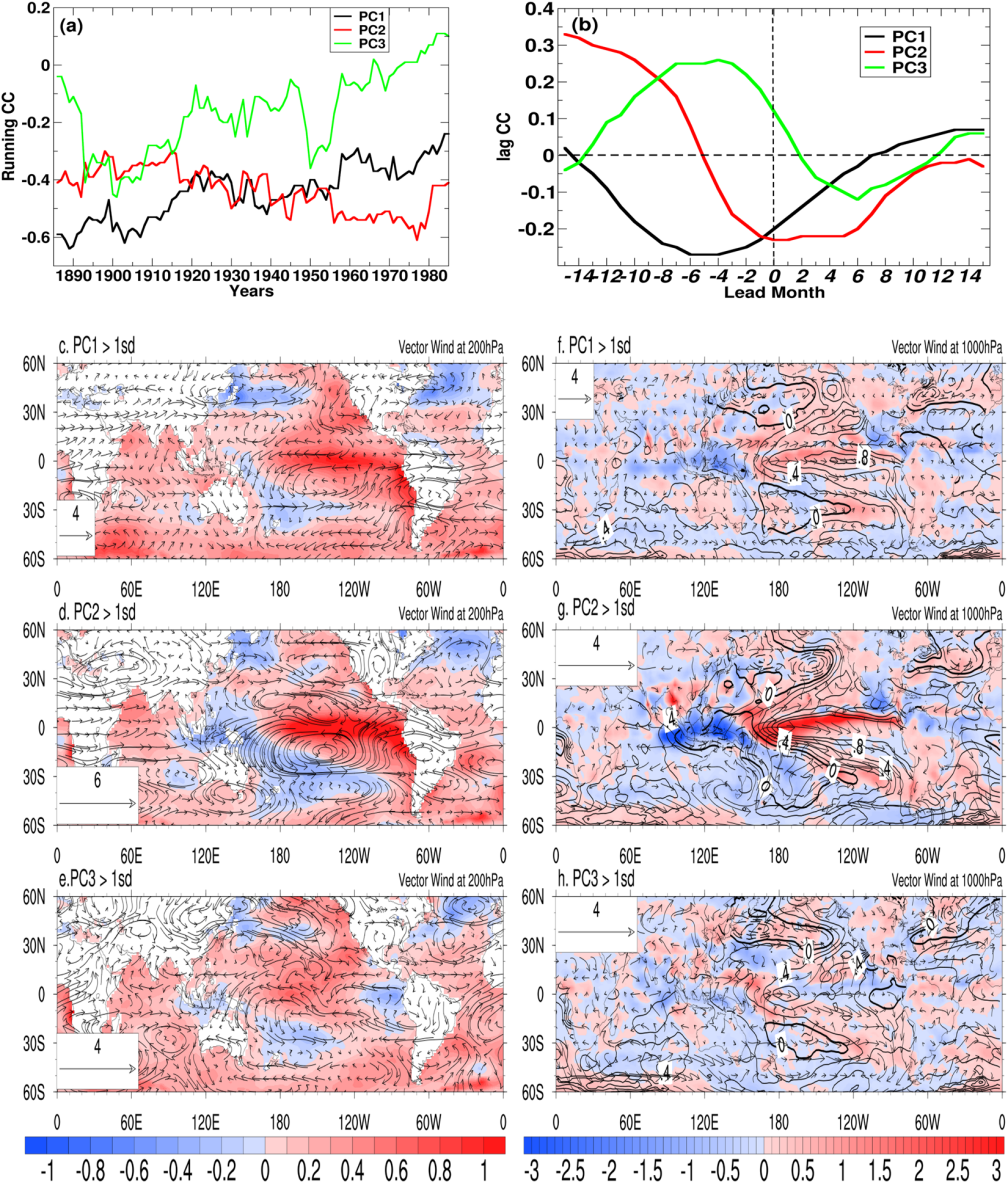
(**a**) 31 year running correlation of PC1, PC2 and PC3 with June-September averaged (JJAS) rainfall over Indian region. (**b**) Lag correlation of PC1, PC2, and PC3 with July rainfall. (**c**) Composite of SST (shaded) and wind at 200hPa for the cases (months) when standardized PC1 is greater than +1 standard deviation (sd) during JJAS; (**d**) same as (**c**) but for PC2; (**e**) same as (**c**) but for PC3. (**f**), composite of SST (contours), rainfall (shaded) and wind at 1000hPa for the cases when standardized PC1 greater than +1 sd. (**g**,**h**) same as (**f**) but showing for cases when PC2 and PC3 greater than +1sd respectively. Signs are adjusted for the standardized PCs so that the values greater (less) than +1 (−1) indicates the El-Nino (La-Nina) case for the SST.

In view of the debate on whether or not the EP or CP type of El Ninos have something to do with the weakening ENSO-Indian monsoon relationship in recent years^31^, we investigate the contribution of the three different modes to the ENSO-Indian monsoon relationship (Fig. 2). The 31-year running mean correlations between ISMR and QQ, QB and DQB modes (i.e. PC1, PC2 and PC3 respectively) show (Fig. 2a) that the correlation between ISMR and QQ mode has a decreasing trend with strong negative correlations in early years decreasing to weak negative correlation in recent years. That with QB mode, on the other hand, has a weak increasing trend with moderate negative correlations in early years going over to strong negative correlations in recent years. With the DQB mode having a strong decreasing trend of correlations with ISMR, the observed decreasing trend in ENSO-ISMR correlations appears to be contributed largely by the QQ mode and the DQB mode (Fig. 2a). Further insight on how the three modes contribute to the ENSO-ISMR can be derived from lead-lag correlations between ISMR and PC1, PC2 and the PC3 (Fig. 2b). The classical lead-lag relationship between ISMR and Nino3.4 (or Nino3)^32^ with peak negative correlation taking place 3–4 months after the peak monsoon seems to come from that with the QB mode while the correlations with the QQ mode and the DQB mode both peak about 4–5 months prior to the peak of the monsoon rainfall (July). It is also interesting to note that the simultaneous correlations between ISMR and the DQB mode is weakly positive while that for the QQ and QB modes is weakly negative. These rather striking differences in correlations between ISMR and the three modes indicate the complexity of the ENSO-Indian monsoon relationship and suggest a cautious interpretation of correlations obtained between ISMR and indices of CP and EP El Ninos^33^. Why the teleconnection between ISMR and the QQ mode (Pacific SST) peaks 4–5 month prior to the peak monsoon rainfall while that with QB mode peaks 3–4 months after the peak monsoon rainfall remains an open question at this point and is subject of future study.

To bring out the pathways for tropical as well as extra-tropical teleconnections associated with the three modes, we construct composite of JJAS SST and 200 hPa wind anomalies for PC1, PC2 and PC3 greater than +1 standard deviation (Fig. 2c–e). Similar composites for SST, precipitation and 1000 hPa wind anomalies are shown in Fig. 2f–h. The boreal summer teleconnections are strongest associated with the QB mode while those associated with the QQ and DQB modes are rather weak. It is interesting to note that the tendency to produce enhanced precipitation in the north-east India and decreased precipitation in the central and western India associated with ENSO (e.g. Fig. 2g) is due to the large scale subsidence (upper level cyclonic circulation) and large scale uplift (upper level anticyclone) as a result of teleconnections with each mode (e.g. Fig. 2d). The corresponding La Nina composites for the modes (for PCs < -one standard deviation) in which the teleconnection patterns simply reverses in sign (Fig. S3) indicates the robustness of the teleconnections. During the boreal winter, the teleconnections are much stronger not only for the QB mode but also for the QQ and DQB modes (Fig. S4). It is also worth noting that one distinct pathway through which the ENSO influences extra-topical climate namely, the Pacific-North-American (PNA) pattern is clearly associated with all the three modes strongest with the QB mode in boreal winter **(**Figs 2 and S4).

### Ocean-atmosphere interaction mechanisms

**Figure 3:**
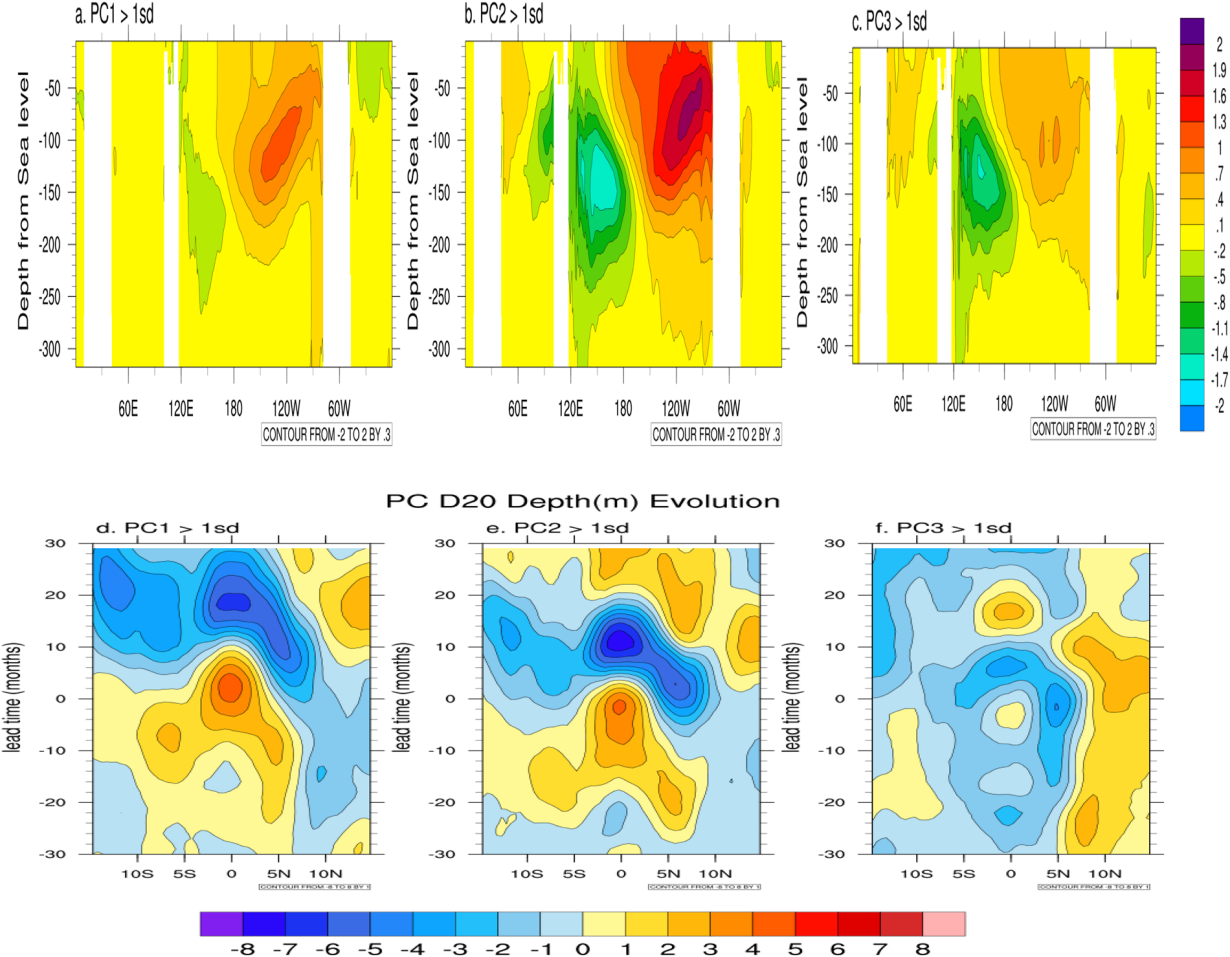
(**a**–**c**) Longitude-depth (2°S–2°N average) cross section of ocean temperature composite for the cases when PC1 > +1sd (standard deviation), PC2 > +1sd and PC3 > +1sd respectively. (**d**–**f**), similar to (**a**–**c**) for latitude-lag plot of the zonal mean 20°C isotherm depth for PCs > +1sd. The SODA reanalysis data (1871–2010) is used for this calculation.

How similar or different is the core ocean-atmosphere interaction mechanism for sustaining the three different modes? The cross section of temperature along the equator as a function of depth at the peak El Ninos (Fig. 3a–c) indicate that for all the three modes a coupled Kelvin wave type thermocline anomalies are seen over the Pacific with strongest anomalies associated with the QB mode with moderate anomalies associated with the QQ mode while very weak anomalies are associated with QQB mode. The thermocline anomalies outcrop in the central Pacific for the QQB mode at lag zero while at the eastern Pacific for the QQB mode as expected from their spatial structures **(**Fig. 1). In both cases the thermocline anomalies propagate eastwards at speeds commensurate with their intrinsic time scales (Figs S5 and S6). Thus, a mechanism similar to a delayed oscillator seems to govern all the modes. From the perspective of mechanisms, the diversity seems to be a result of varied contributions of the three different modes to a specific event.

At the heart of the quasi-cyclic ENSO evolution is the movement of the upper-ocean warm water volume (WWV) to and from the equatorial thermocline, a process known as WWV recharge–discharge^34,35^. The composite anomalies of zonal mean depth of 20 °C isotherm as a function of latitude around peak El Ninos from 30 month lag to 30 month lead (Fig. 3d–f) for the three different modes to indicate how the recharge-discharge seem to work for the QQ mode, the QB mode and the QB mode at their individual time scales. Specifically, for the QB mode (Fig. 3e), the equatorial thermocline depth anomalies peak (recharged stage) before the peak of mode (PC2) indicating that the thermocline feedback mechanism or the recharge/discharge model of ENSO development is at work for this mode. The recharge/discharge model is also applicable for the QQ mode (Fig. 3d) even though the time scale for the mode is quasi-four year rather than quasi-two year for the QB mode. However, there is an important difference. The equatorial thermocline depth anomalies peak (recharged stage) after the peak of the mode (PC1) by 3–4 months. This indicates that while the thermocline feedback and equatorial dynamics is the dominant mechanism for the QB mode, zonal advection (possibly involving off-equatorial dynamics) appears to make a significant contribution to the buildup of equatorial thermocline anomalies for the QQ mode. The differences in the evolutionary pattern of Pacific SST for the two modes (Fig. S2) as result of similar and yet differing air-sea interactions involved may be responsible for the differences in the teleconnections of the two modes with ISMR (Fig. 2b). On the other hand, the picture for the QQB mode (Fig. 3f) is different where we see that a very weak recharge-discharge operates on a biennial time scale on the top of a background thermocline that varies on a decadal time scale possibly involving subtropical influence. Therefore, the QQB mode is important in providing insight to the diversity of mechanisms associated with different ENSO events.

### Nonlinearity and predictability

**Figure 4:**
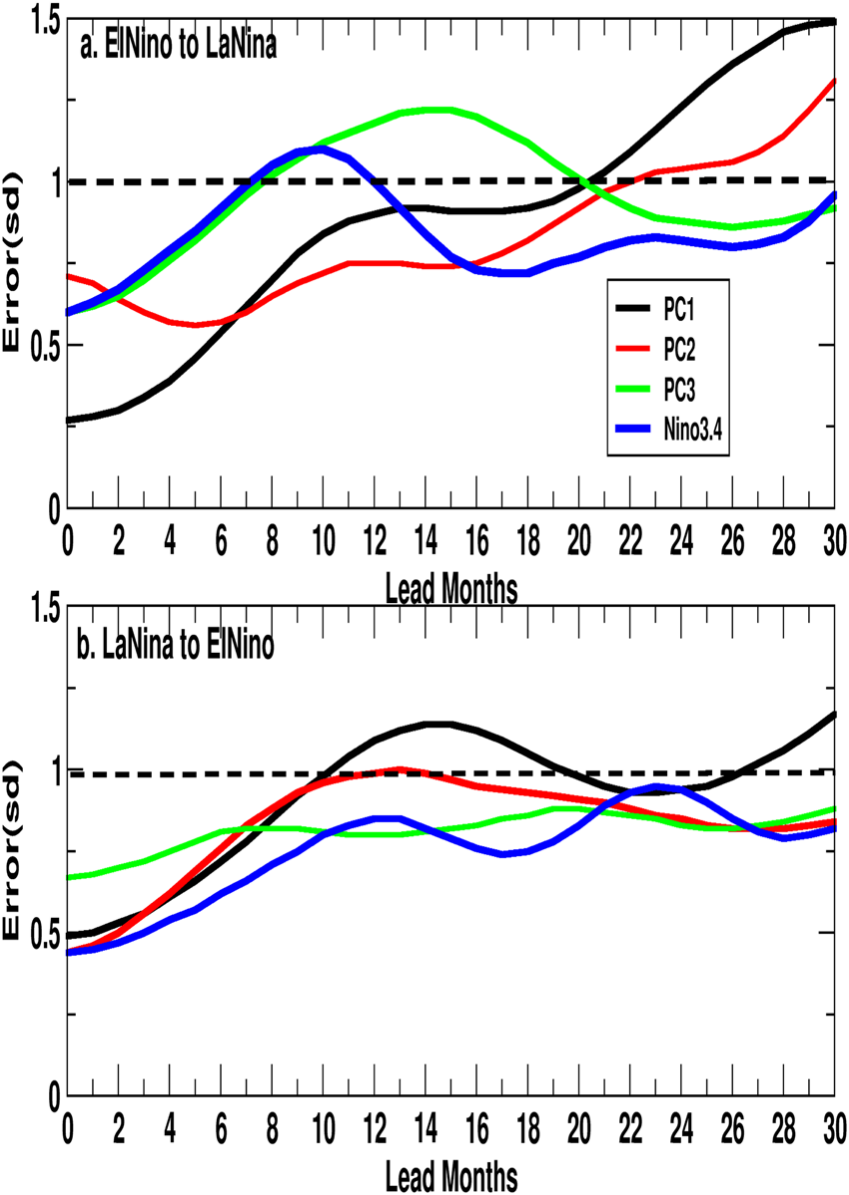
(**a**) Growth of errors from peak El Nino to La Nina for Nino3.4, PC1, PC2, PC3; (**b**) Same as (**a**) but for peak La Nina to El Nino. Errors are defined as the standard deviation of the Niño index from all the sample days clustered at each lead time starting from the peak day.

Early estimates of potential predictability of the ENSO^9,10^ did not recognize the existence of the different types of El Ninos. Therefore, it is natural that attempts have been made to estimate the predictability of the CP and EP types of El Ninos^36,37^. Hence, it is important to understand the limits of predictability of the three different modes to gain insight on limits of predictability of diversity of the ENSO. We recognize the nonlinearity of the ENSO manifesting in the asymmetry between the amplitude and evolutionary characteristics of the positive and the negative phases. As a consequence, the growth rate of errors may be different going from a peak El Nino to a La Nina and or from a peak La Nina to an El Nino. By using a technique developed for estimating the predictability limits of the monsoon sub-seasonal oscillations^38^ (also refer method section), the estimate the growth of initial errors going from El Nino to La Nina in observations (the Nino3.4 SST time series) as well as from PC1, PC2 and PC3 are made (Fig. 4a) (see Methods). Similar estimates of growth of initial errors going from La Nina to El Nino are shown in Fig. 4b. It is evident that the ‘initial error’ is already large, as the method has no control on the ‘initial error’ defined as the standard deviation of the amplitude of a chosen phase (e.g. peak El Nino, Fig. 4a or peak La Nina, Fig. 4b), making the growth of errors in the nonlinear regime. While keeping this caveat in mind, we note that the growth of errors of Nino3.4 SST is fast from El Nino to La Nina limiting predictability to only about 8 months while that from La Nina to El Nino is slow with predictability extending to at least 24 months. The growth of errors in the two types of transitions is consistent with the recharge process building up to an El Nino being much slower than the discharge process from an El Nino to a La Nina (Fig. 3). The combined EEOF analysis brings out differences in lead-lag evolution of these modes. The differences in ENSO evolutionary modes related to equatorial and off-equatorial buildup of heating suggest that these modes would evolve in different ways real situations and predictability of these modes would differ. Interestingly, the error growth in both transitions for Nino3.4 SST is very similar to that of the DQB mode indicating that in observations (and probably in predictions as well), the growth of errors is dominated by this mode. Thus, models must simulate this mode also with fidelity, even though it represents smaller amount of inter-annual variance than the other two modes. It is also interesting to note that growth of errors for QQ and QB modes are slow from El Nino to La Nina with predictability extending to about 20 months while that is fast from La Nina to El Nino limiting predictability to about 10 months.

### ENSO diversity

**Figure 5:**
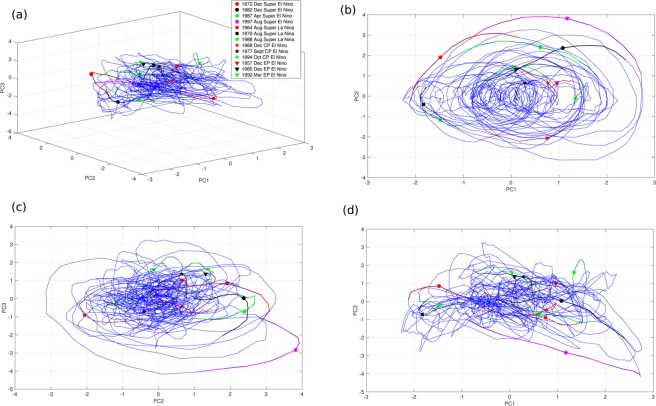
(**a**) Evolution of the state of ENSO in the 3-D phase space of PC1, PC2 and PC3 between 1854 and 2004. (**b**–**d**) Same evolution but projected on 2-D phase spaces: (PC1, PC2), (PC2, PC3), (PC3, PC1).

**Figure 6:**
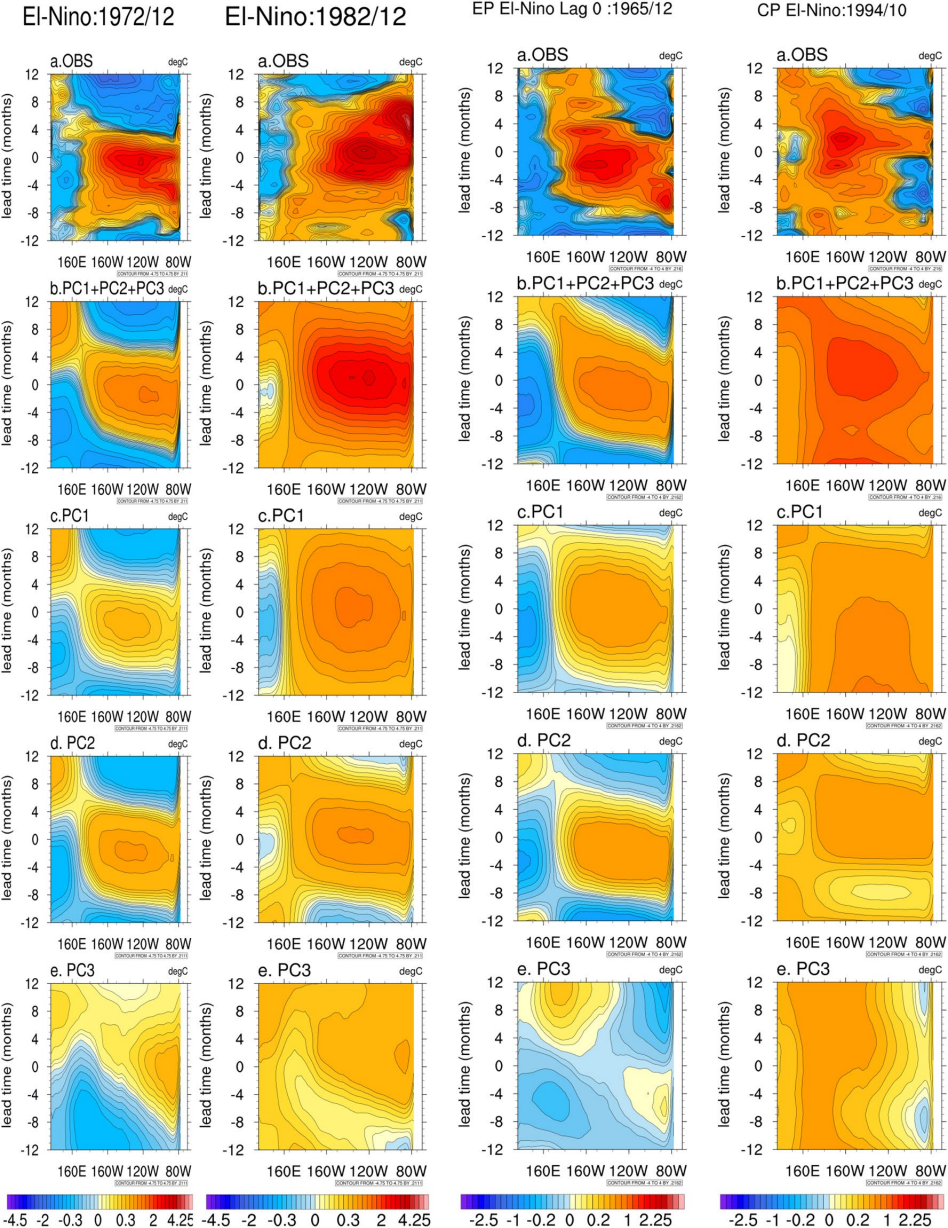
Time-longitude section (10_S–10_N average) of different types of El-Niños and their reconstruction based on EEOFs. First two column shows super EL Niños (1972, 1982), the third column shows an EP El Nino (1965) and the fourth column shows a CP El Nino (1994) from Observations (**a**) and their reconstruction from EOF1, PC1) + (EEOF2, PC2) + (EEOF3, PC3***)*** (**b**),(EEOF1, PC1) (**c**), (EEOF2, PC2) (**d**) and (EEOF3, PC3) (**e**) respectively. The reconstruction is based on EEOFs and PCs of these modes (refer text). Lag 0 is the peak month taken multivariate ENSO index data. Refer^56^ for the years selected for the study. The shading intervals are unevenly spaced.

As the ENSO ‘slow manifold’ is fully defined by these three modes, the monthly values of the three PCs from 1854 to 2004 in the three dimensional phase space defined by PC1, PC2 and PC3 defines the evolution of the state of the ENSO during the period (Fig. 5). Diversity of ENSO (e.g. super El Ninos, Super La Ninas, EP El Ninos and CP El Ninos) essentially results from varying contributions from the three modes to a particular event. Evolution of 12 months around peaks of 4 super El Ninos, 3 super La Ninas, 3 CP El Ninos and 3 EP El Ninos are marked in Fig. 5 (see Method for selection of types of ENSO) to get a sense of how they are contributed by the three modes. We reconstructed these events only with the three PCs from 12 months prior to 12 months after the peak month of each event. Time-longitude section of two super El Ninos, one EP El Nino and a CP El Nino from observations and reconstructed from all three PCs with contributions each are illustrated in Fig. 6. While some high frequency processes (period shorter than a year) does contribute to the observed amplitude and spatial location of the maximum (as seen in Nino3.4 cross section in Fig. 6), it is rather remarkable that the reconstruction with the three modes captures the timing of the transitions (both from +ve to −ve and from −ve to +ve) as well the timing of the maximum quite accurately. Reconstruction of the other events (Fig. S7) also support this conclusion that the three modes are sufficient to describe the space-time structure of all the diverse types of ENSO.

To further illustrate the efficacy of the reconstruction with the three leading modes, the modal reconstruction of Nino3.4. Index is shown in Fig. S8a from one SST dataset. The reconstruction of Nino3.4 and the modal evolution show how the different modes contributed to the evolution of El-Nino, La Nina or neutral conditions. Stronger El-Nino or La Nina cases show in phase contribution of all the three modes, while in certain situations, they acted out of phase and reduced the Nino3.4 index amplitude. The difference may be partly due to un-coupled variability in Nino3.4 and contributions from high frequency noise. As against Nino3.4, the extended MEI index^25^ is a coupled ENSO index using EOF analysis. Comparison of our reconstruction of Nino3.4 with MEI Index (Fig. S8b) indicates much improved correspondence indicating near 70% variance explained by the three leading mode.

Is there any pattern to indicate the combinations of the three modes that determines a particular type of ENSO as identified in several literatures [e.g.^56^]? The normalized values of the three PCs for the events mentioned above indicate that the QB mode seems to be a determining factor for the super El Ninos with its sign reversing from super El Ninos to super La Ninas. In addition, both QQ mode and DQB mode being in the negative side ensures a super La Nina. However, no clear pattern is apparent for contributions of the QQ mode and DQB mode to the super El Nino. Similarly, no distinguishing pattern in terms of contribution of the three modes seems to emerge that distinguishes an EP El Nino from a CP El Nino. This may be because, what we have been considering to be the CP mode so far (our lag 0 QQB mode) is not a stationary pattern but has an evolutionary history (see EEO3, Fig. S2).

### Modal description of simulated ENSO

Notwithstanding notable progress made by climate models, they still suffer from significant biases in simulating the amplitude and evolutionary characteristics of the ENSO^39^ limiting the skill of ENSO predictions mentioned earlier. Examination of the models’ biases in simulating and predicting the three leading modes may not only provide understanding about their limitations in simulating and predicting the ENSO diversity but also provide clue towards improvements. In order to illustrate the application of this framework on model simulations, we examine the ENSO in a relatively long simulation of a climate model. For this purpose, we selected a 500-year long pre-industrial control run of the GFDL CM3 coupled model^40^. The leading three EEOFs based on monthly SST and SLP data for 200 years using lagged copies up to 18 months (Fig. S9) indicate that the first EEOF is like the observed QQ mode but with slightly higher frequency (period peaking around 33 months). The dominant period associated with the EEOF2 is also approximately 33 months similar to EEOF1 but with a distinct evolutionary history (Fig. S9). Also, it is noted that the variances explained by the first EEOF (10.5%) and that by the second EEOF (9.3%) are too close to each other. Thus, the model instead of simulating a distinct QB mode, simulates another shade of the QQB mode. The EEOF3 is dominated by a quasi-biennial frequency but the model fails to simulate the decadal modulation of the QB mode. The evolution history of the EEOFs (Fig. S10) indicate the EEOF1 has the spatially stationary evolution like observed with longer period while the other two has evolution with anomalies starting in the eastern Pacific expanding westwards. How these biases in simulating the three modes translate to biases in simulating the teleconnections and predictability is shown in Fig. S11 (see Supplementary Material for details).

### Robustness of the leading modes

The analysis of modal representation of ENSO uses the exploratory data tools like EEOF analysis. In order to confirm the physical robustness of the modes, we use multiple SST data to confirm the existence of the leading three modes. The additional independent SST reanalysis data used are the Kaplan SST^41^, HADLSST^42^ and COBE SST2^43^. HADSLP^44^ data is used as the common SLP data for all these modes as no other long term SLP data is available. The spectra of PCs of the three leading combined EEOFs are shown in Fig. S12 for different SST data sets together with the corresponding EEOFs at lag 0 of SST and SLP in Fig. S13. Modal distribution of variance remains in the similar order. The presence of three leading modes with spectral signature in the quasi decadal scale, quasi quadrennial scale and quasi biennial scale with their unique spatial patterns in all data sets indicates robustness of the three leading modes and may be fundamental to understanding ENSO dynamics. Some reorganization in the spectral variance observed may be expected as the percentage of variance explained by the leading modes depends on the spatial structure and variance of the high frequency ‘noise’ expected to be different in different SST reanalysis data sets.

## Conclusions

Understanding ENSO diversity appears to be the next milestone of ENSO research^16^, essential for advancing the skill of ENSO predictions. Due to its ‘diversity ‘and ‘complexity’, the ENSO cannot be represented by just one mode or a simple physical process. Are there some leading modes that represent the whole spectrum of ENSO variability and could the ENSO diversity be realized and quantitatively understood in terms of these leading modes? In this study, we answer that question in affirmative and show that indeed the ENSO *slow manifold* could be represented by three spectrally well separated physical modes, a quasi-quadrennial mode (QQ), a quasi-biennial mode (QB) and decadal modulation of quasi-biennial mode (DQB), that are distinct not only in temporal domain, but also in temporal evolution of the spatial structures. Further, it is demonstrated that reconstruction based on the three modes describe the diverse ENSO types in their amplitude and temporal evolution. It is, therefore, imperative that climate models simulate the three leading modes with fidelity to be able to simulate ENSO diversity and hence improved ENSO prediction.

With the differences in temporal characteristics and spatial patterns, it is natural that teleconnections of ENSO modes with various regional climates would be different. Thus, this framework also provides a platform to understand ENSO-regional climate teleconnections as a consequence of teleconnections with individual modes. We illustrate this with teleconnection between ENSO and Indian summer monsoon. The global teleconnection pathways during boreal summer and winter associated with the three modes also provide insight on the origin of global ENSO teleconnections. The nonlinearity of ENSO^45,46^ (asymmetry between positive and negative phases) leads to faster growth of errors going from El Nino to La Nina while slower growth of errors going from La Nina to El Nino resulting in different limits on predictability for the two transitions. While the leading modes are linear decomposition of the ENSO mode, they have their individual asymmetry in El Nino and La Nina phases. Different nonlinearity associated with the three modes and the asymmetry in the spatial pattern associated with the peak El-Nino and peak La-Nina due to phase difference in temporal evolution as shown in the lag regression relationship (e.g. Fig. 3d,e,f), leads to differences in the error growths for the three modes associated with the two transitions providing insight towards the observed limit on predictability.

Finally, using the long and reasonable simulation of ENSO by a climate model, we demonstrate that the framework presented here provides a useful way to diagnose biases of a model in simulating the ENSO and its predictability. The nature of biases in representation of the leading modes and their teleconnections provide useful clues towards improvement of the model, for improved simulation of the ENSO diversity and improvement of skill of ENSO prediction.

## Methods

The study uses the extended empirical orthogonal function (EEOF) analysis^47^ to isolate the three modes (principal components) of oscillation based on two variables: sea level pressure (SLP) and sea surface temperature (SST) data. Combined EEOF analysis can reveal the underlying coupled modes using SST and SLP at the same time it can effectively remove the temporal auto and cross correlation in addition to the spatial auto and cross correlation used in traditional empirical orthogonal function (EOF) analysis^47^. EEOF analysis has successfully isolated the summer monsoon intraseasonal mode^48,49^. This combined (C-EEOF) EOF analysis has been applied to isolate intraseasonal oscillation like Madden Julian Oscillation (MJO)^50^. This study extends the idea of traditional EOF analysis for two variables (SST and SLP) to the combined EEOF. In order to create the covariance matrix, we use the same method as Wheeler and Hendon, 2004 that uses three variables dimensions. In this case, the schematic of the data matrix which has SST and SLP as two variables with ***n*** time points, ***m*** lag and ***p*** space points that is used to construct the covariance matrix is shown here:

$$X=|(SST(row=\mathrm{1..}n-m,column=1\ldots p\ast m|SLP(row=\mathrm{1..}n-m,column=1\ldots p\ast m)|$$

Thus each lag is appended side by side as columns in the extended data matrix. Now the transpose of **X**: **X**^**T**^ **is** created and standard eigen analysis is then performed with identification of the eigenvectors (aka EOFs) and the principal components (PCs) stratified in a descending order based on fraction of variances explained by each eigenmodes.

We have used 151 (1854–2004) years of monthly reanalysis of SST and SLP data i.e. **n** = 151 * 12. The SST data used is the NOAA Extended Reconstructed SST V5^51^ and is downloaded from download link provided in https://www.esrl.noaa.gov/psd/data/gridded/data.noaa.ersst.v5.html. The SLP data is taken from Hadley Centre and is known as HADSLP2 data^44^ and is downloaded from https://www.metoffice.gov.uk/hadobs/hadslp2/. SLP data is re-gridded to SST grid (89 latitude X 180 longitude) before the EEOF analysis. A few standard preprocessing before doing EEOF analysis is done using SST and SLP: (a) first the climatology of the whole data is removed to create monthly anomalies, (b) trend from the data is removed and, (c) data at each grid point is weighed by cosine of latitude.

Lag ***m*** is chosen as 18 months and space points (***p***) are chosen for tropical Pacific (30°S–30°N, 100°E–100°W). m = 18 is chosen with some experimentation which include one quarter cycle of ENSO periodicity (~60 months). Results are consistent if we use say 15 months lag but the statistical significance of the decadal mode is clearer. Also, we checked using Eq. 24 of North *et al*.^52^, that these modes are significant and non-degenerate.

To get insight into the ocean-atmosphere interaction associated with the modes, in addition to the SST data, we also examine sub-surface ocean temperatures from the SODA^53^. The SODA v2.2.4 Data is downloaded from http://apdrc.soest.hawaii.edu/data/data.php which is maintained by Asia-Pacific Data Research Center and is a part of the International Pacific Research Center at the University of Hawai’i at Mānoa, funded in part by the National Oceanic and Atmospheric Administration (NOAA). The SODA subsurface ocean temperature and the depth of 20 °C isotherm is calculated and plotted for verification of the SST data.

The climate model data used here to compare with the reanalysis data is the GFDL CM3 model^40^ run with preindustrial (1860) control greenhouse forcing based on CMIP5 protocol^54^. The data is downloaded from: http://nomads.gfdl.noaa.gov:8080/DataPortal/cmip5.jsp and more details about the GFDL CM3 run are given in https://www.gfdl.noaa.gov/coupled-physical-model-cm3/. Model output has 500 years of monthly data and have 144 longitude and 90 latitudes. For EEOF analysis we skip first 100 years of data and did an EEOF analysis on next 200 years of data. We followed the exact similar procedure as with the reanalysis SST and SLP data to derive the EEOFs and PCs based on model SLP and SST. Subsequent analysis is followed using the same method as with reanalysis SLP and SST.

In order to understand the ENSO diversity for different *flavors* of El-Nino, we also provide a reconstruction of few cases of super El-Ninos, Central Pacific (CP) type El-Ninos and Eastern Pacific (EP) type EL-Ninos. The reconstruction of SST data from the EEOFs and PCs for the ***ith*** mode is as follows^55^:

$$SS{T}_{i}(t,lat,lon)=\frac{1}{m}\times \mathop{\sum }\limits_{lag=1}^{m}{\theta }_{i}\times EEO{F}_{i}(lag,lat,lon)\times P{C}_{i}(t-lag)$$

where *θ*_*i*_ is the eigenvalue of the ***ith*** mode, ***m*** is the lag used i.e. 18 months. We have shown separately the reconstruction patterns for first three EEOF modes (*i* = 1, 2, 3) and the sum of the modes (1 + 2 + 3).

Finally we estimated growth of error in the following way: first we identified the all the peak EL-Nino and La-Nina from the nino 3 data index. Then, for all the identified peak events (day 0), we calculated the standard deviation of day 0, day 1 …etc. based on the number of identified maxima (El-Nino) and minima (La-Nina). A plot of amplitude of standard deviation as a function of lead-time would give the estimate of event-to-event variability. This event-to-event variability is a measure of error growth at any lead-time^38^.

## Supplementary information

Supplementary Figures

Supplementary Figure 5

Supplementary Figure 6
