# Supplementary material for: A Modal Rendition of ENSO Diversity
Source: Sci Rep. 2019 Sep 30;9:14014. doi: 10.1038/s41598-019-50409-4 (PMC6768998; doi:10.1038/s41598-019-50409-4)
Supplement: Supplementary file 1 — Supplementary Figures [file 41598_2019_50409_MOESM1_ESM.pdf]

A Modal Rendition of ENSO Diversity

Rajib Chattopadhyay[1], S. Dixit[1] and B. N. Goswami[2]#

[1]Indian Institute of Tropical Meteorology, Pune, India

[2]Cotton University, Guwahati,India

[#]Corresponding Author email: bhupengoswami100@gmail.com

Supplementary Figures

Figure Caption

Figure S1 The time series of the first three principal components (PC1,PC2 and PC3) derived by projecting the EEOFs onto the data (covariance matrix). The plot for each PC series is split into two panels, with the top panel depicting evolution from 1854-1934 and the bottom panel showing evolution from 1935-2004.

Figure S2 Time evolution of combined EEOFs of SST and SLP for the Pacific (30°S-30°N;90°E-90°W) for all the 18 lags which is used to construct the covariance matrix(see text).(a)-(c) shows the SST EEOFs (EEOF1,EEOF2 and EEOF3) while (d)-(f) shows the SLP EEOFs. The lag for each EEOFs are mentioned at the top of each sub panels. The variance explained by each mode is also mentioned at the top of each panels.

Figure S3 (a)Composite of SST (contours), rainfall (shaded) and vector wind at 1000hPa for the June-September (JJAS) averaged cases (months) when standardized PC1 is less than -1 standard deviation(sd); (b) same as (a)but for PC2; (c) same as (a) but for PC3. (d)-(f) same as (a)-(c) but plot showing the composite of SST(shaded) and vector wind at 200hPa. Signs of PCs are adjusted for the standardized PCs so that the values less than -1 indicates the LaNina case for the SST.

Figure S4 Same as Fig. S3 but for the El Nino composite cases cases (i.e. standardized PCs greater than 1 standard deviation),averaged for the November-January (NDJ) months.

Figure S5 Animation plots showing the composite evolution of depth-longitude profile of water temperature (from SODA during El-Nino from lag -15 to lag +15. Lag 0 is the month when the respective PCs (PC1,PC2,PC3), mentioned at top of each panels attain maximum value depicting the peak El-Nino for that mode. Signs of PCs are adjusted for the standardized PCs so that the values greater (less) than +1 (-1) indicates the El-Nino (LaNina) case for the SST. The animation is loaded as a separate animation plot in a supplementary powerpoint file.

Figure S6 Same as Fig.S5 but for LaNina.The animation is loaded as a separate animation plot in a supplementary powerpoint file.

Figure S7 Same as Fig.6 but showing few other cases of time-longitude section(10°S-10°N average) of different types of El-Ninos and their reconstruction based on EEOFs. The shading intervals are unevenly spaced.

Figure S8 (a)Nino3.4 SST from ERSSTv5 (black bar) and reconstruction of Nino3.4 SST index based on reconstruction of mode 1, mode 2 and mode 3 and mode (1+2+3) using the EEOFs and corresponding PCs.(b)Panel shows extended MEI index (black bars) and the same reconstruction modes as in (a). Legends of indices and reconstruction modes are mentioned at the top of each panel. Also quoted the linear correlation (cc) values in each panels.

Figure S9 EEOFs and power spectra of PCs from GFDL long runs . (a)-(e)shows the EEOF patterns at lag 0 and (g)-(i) shows the power spectra of each of PC1,PC2 and PC3 respectively (cf.Fig.1). The combined EEOF analysis is based on monthly data of SST and SLP from 200 years of GFDL preindustrial control (PI) run with the 1850 greenhouse forcing as per as CMIP5 protocols. Monthly data from 200 years after the first hundred years have bemused for the EEOF analysis. Preprocessing and other steps for doing the EEOF analysis from the GFDL data are similar to what is done for EEOF analysis based on HADSLP and ERSST reanalysis (see text).

Figure S10 Evolutionary history of the three GFDL simulated EEOFs for all the 18 lags as considered in the last plot.

Figure S11 (a) 31 year running correlation between PC1, PC2 and PC3 and June-September averaged (JJAS) rainfall over the Indian region for the GFDL simulation.(b)Error growth from El Nino to La-Nina for GFDL simulated Nino3.4 and the three PCs from EEOF analysis of GFDL runs (cf. Fig. S12). (c)Same as (b) but for transitions from peak La Nina to El Nino. Fig.2(a) and Fig.4a-b may be referred for a comparison with observation/reanalysis.

Figure S12 Power spectra of PCs computed from multiple SST dataset.Each SST dataset is mentioned at the top of each panel.

Figure S13 First three EEOFs (numbered top to bottom) of SST and SLP based Kaplan, HADLSST and COBE SST2.

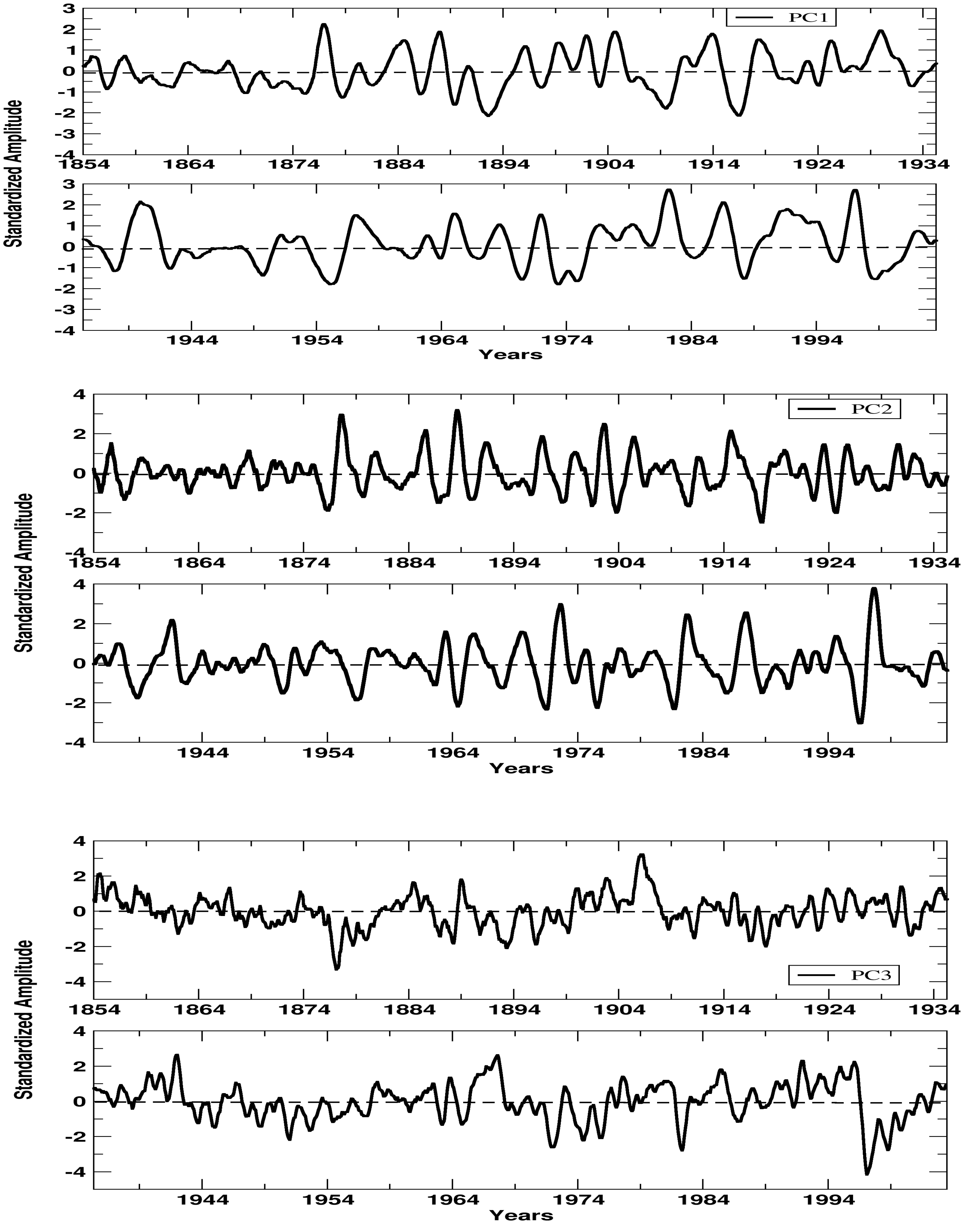

Figure S1: The time series of the first three principal components (PC1,PC2 and PC3) derived by projecting the EEOFs onto the data (covariance matrix). The plot for each PC series is split into two panels, with the top panel depicting evolution from 1854-1934 and the bottom panel showing evolution from 1935-2004.

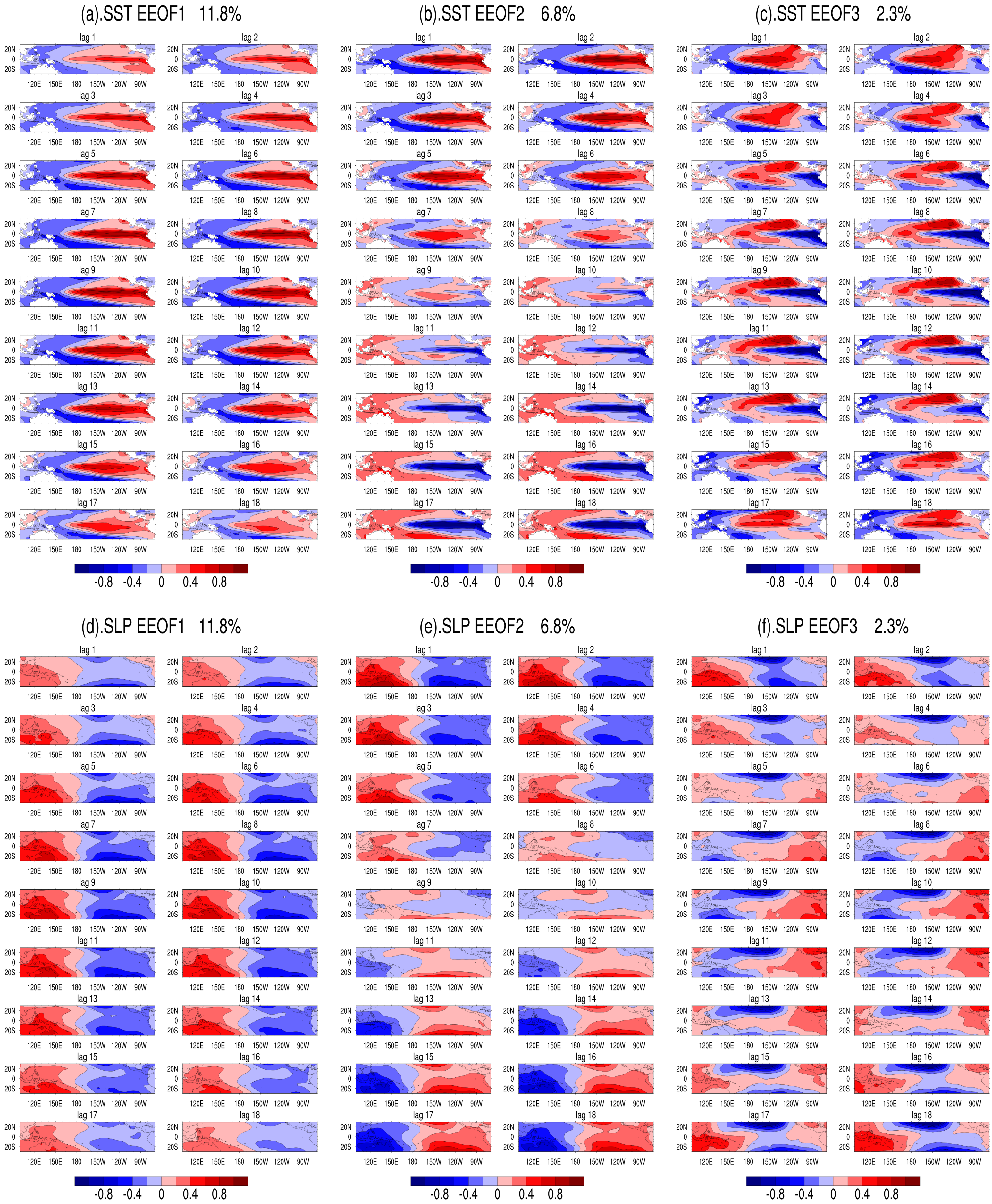

Figure S2: Time evolution of combined EEOFs of SST and SLP for the Pacific (30°S-30°N;90°E-90°W) for all the 18 lags which is used to construct the covariance matrix(see text).(a)-(c) shows the SST EEOFs (EEOF1,EEOF2 and EEOF3) while (d)-(f) shows the SLP EEOFs. The lag for each EEOFs are mentioned at the top of each sub panels. The variance explained by each mode is also mentioned at the top of each panels.

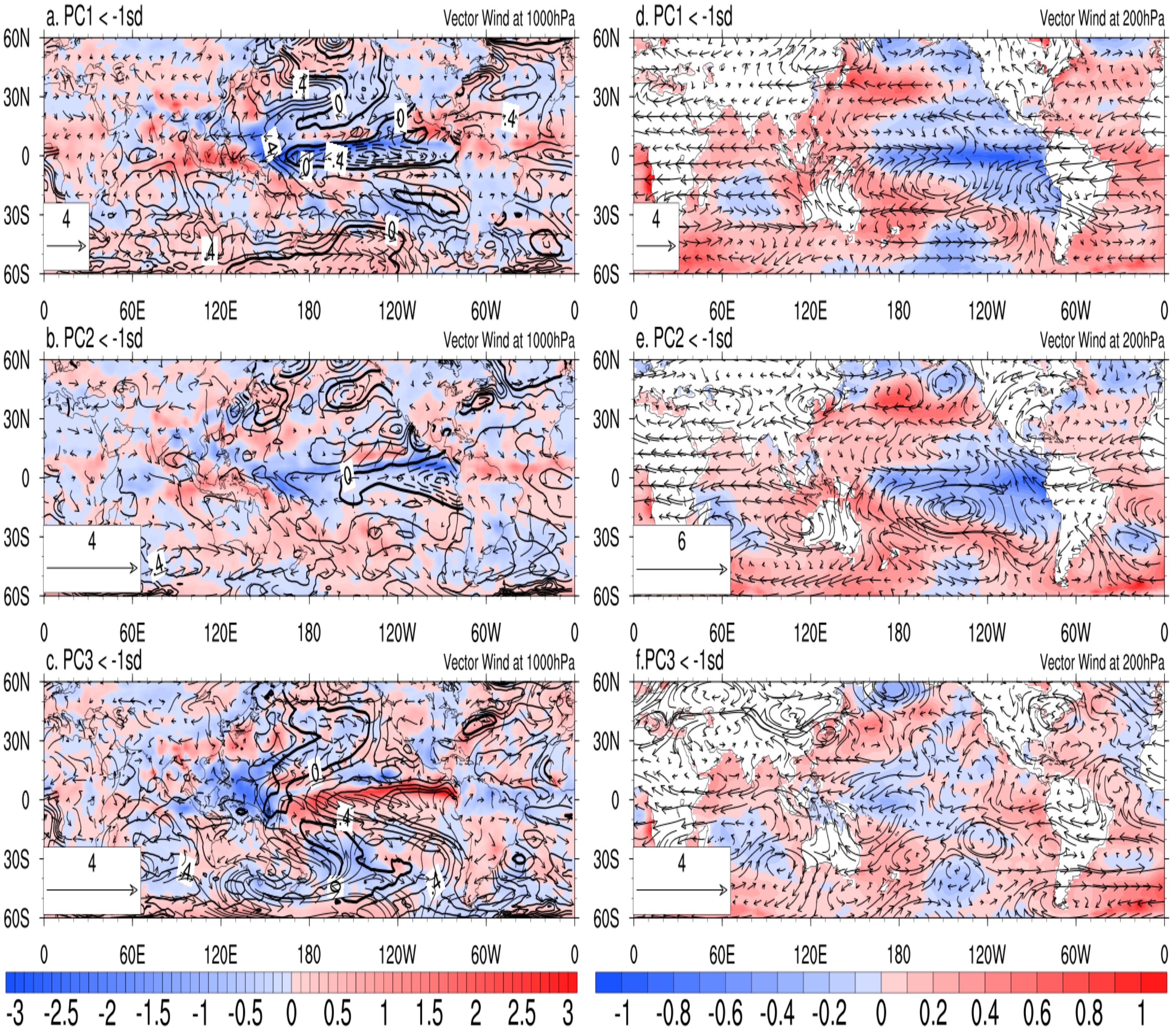

Figure S3: (a)Composite of SST (contours), rainfall (shaded) and vector wind at 1000hPa for the June-September (JJAS) averaged cases (months) when standardized PC1 is less than -1 standard deviation(sd); (b) same as (a)but for PC2; (c) same as (a) but for PC3. (d)-(f) same as (a)-(c) but plot showing the composite of SST(shaded) and vector wind at 200hPa. Signs of PCs are adjusted for the standardized PCs so that the values less than -1 indicates the LaNina case for the SST.

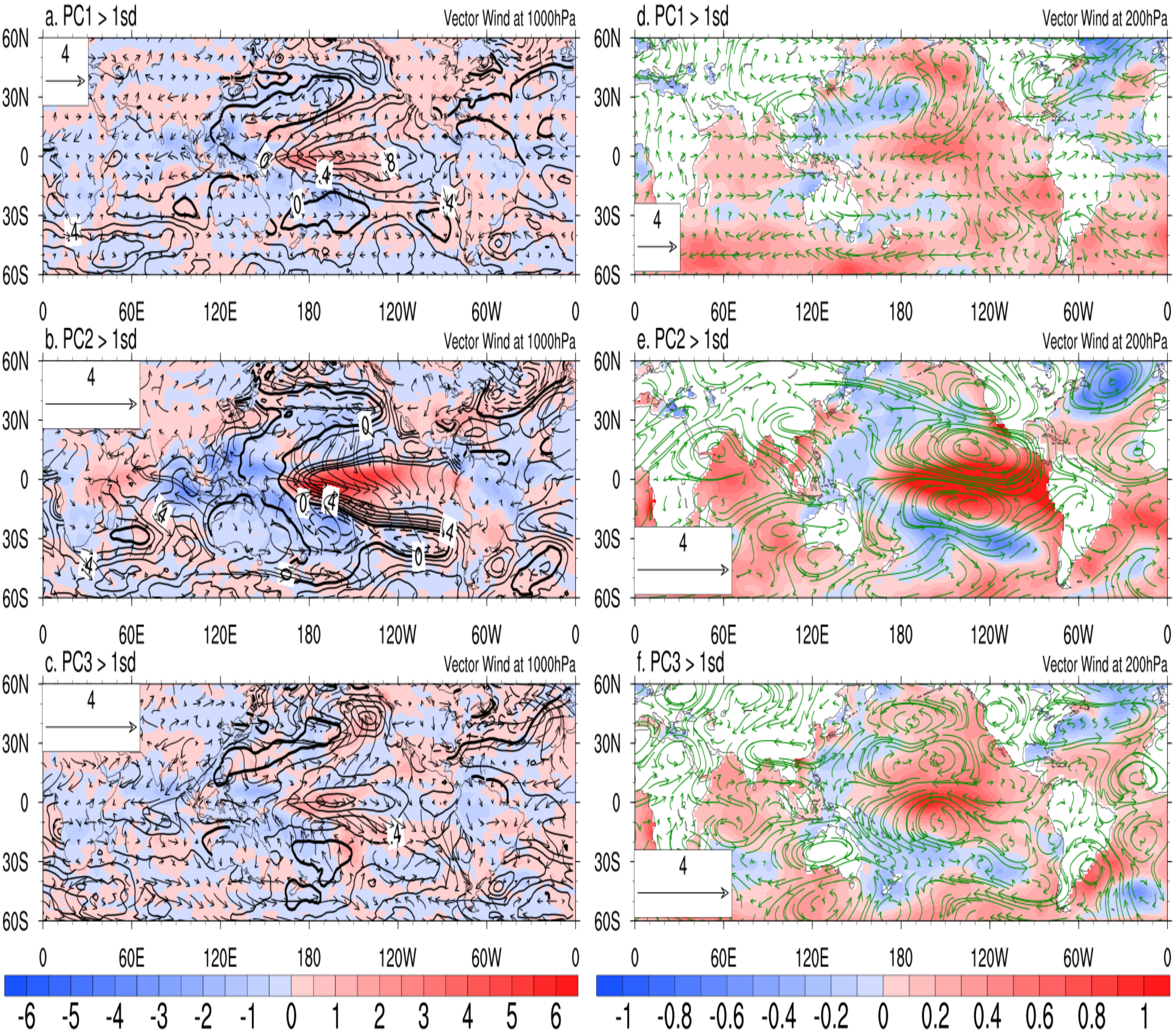

Figure S4: Same as Fig. S3 but for the El Nino composite cases cases (i.e. standardized PCs greater than 1 standard deviation),averaged for the November-January (NDJ) months.

Figure S5: Animation plots showing the composite evolution of depth-longitude profile of water temperature (from SODA during El-Nino from lag -15 to lag +15. Lag 0 is the month when the respective PCs (PC1,PC2,PC3), mentioned at top of each panels attain maximum value depicting the peak El-Nino for that mode. Signs of PCs are adjusted for the standardized PCs so that the values greater (less) than +1 (-1) indicates the ElNino (LaNina) case for the SST. The animation is loaded as a separate animation plot in a supplementary powerpoint file.

Figure S6: Same as Fig.S5 but for LaNina.The animation is loaded as a separate animation plot in a supplementary powerpoint file.

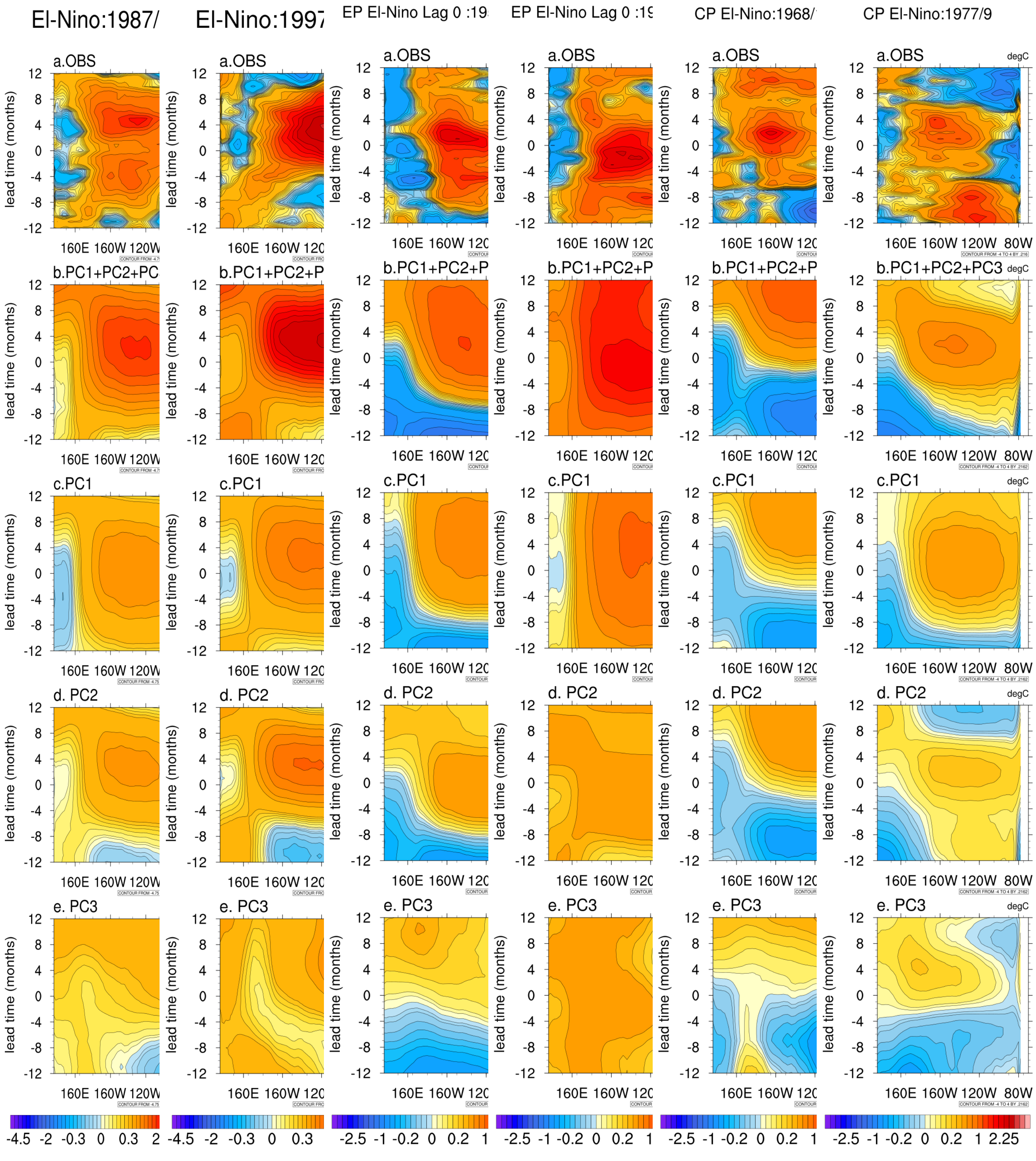

Figure S7: Same as Fig.6 but showing few other cases of time-longitude section(10°S-10°N average) of different types of El-Ninos and their reconstruction based on EEOFs. The shading intervals are unevenly spaced.

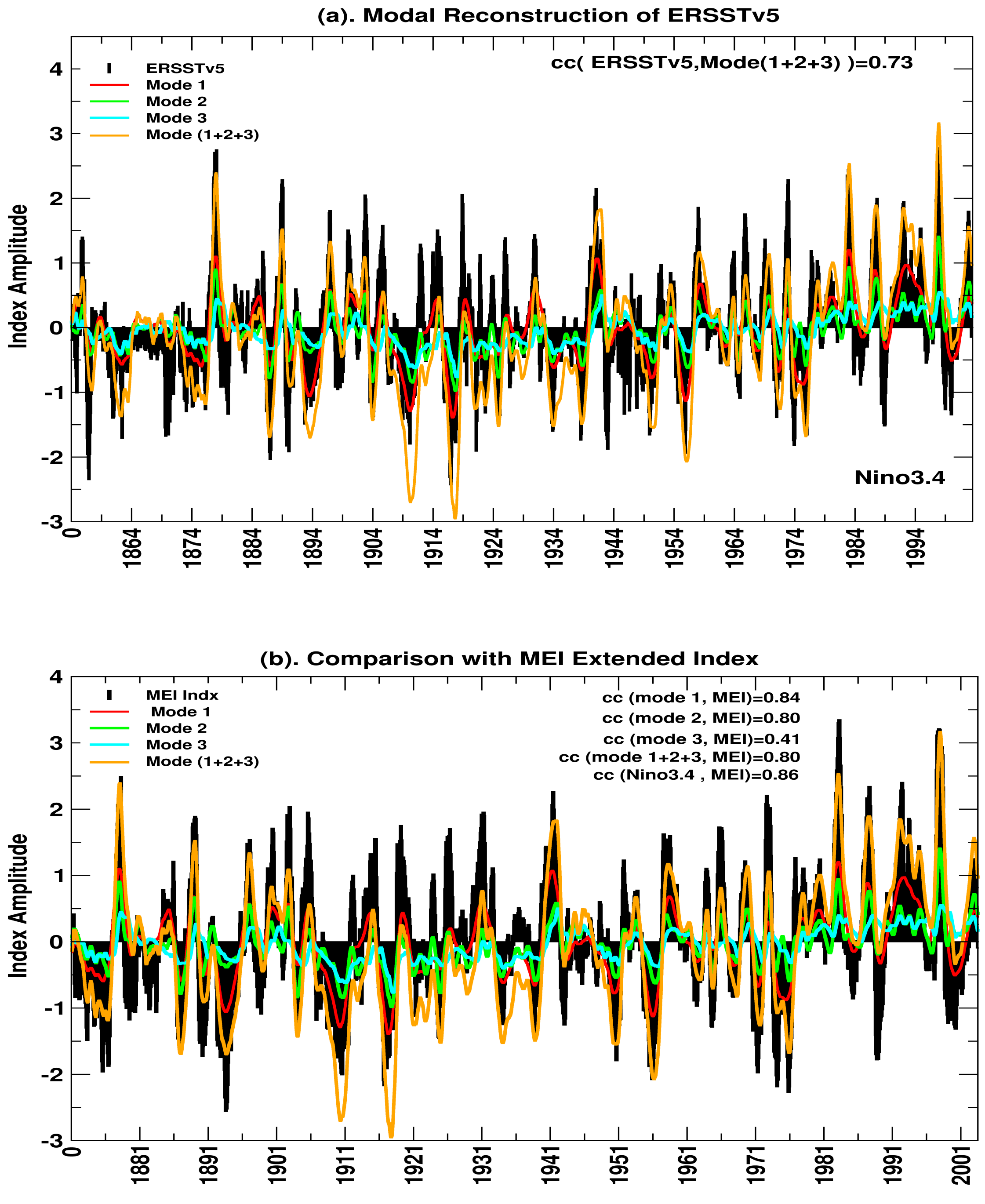

Figure S8: (a)Nino3.4 SST from ERSSTv5 (black bar) and reconstruction of Nino3.4 SST index based on reconstruction of mode 1, mode 2 and mode 3 and mode (1+2+3) using the EEOFs and corresponding PCs.(b)Panel shows extended MEI index (black bars) and the same reconstruction modes as in (a). Legends of indices and reconstruction modes are mentioned at the top of each panel. Also quoted the linear correlation (cc) values in each panels.

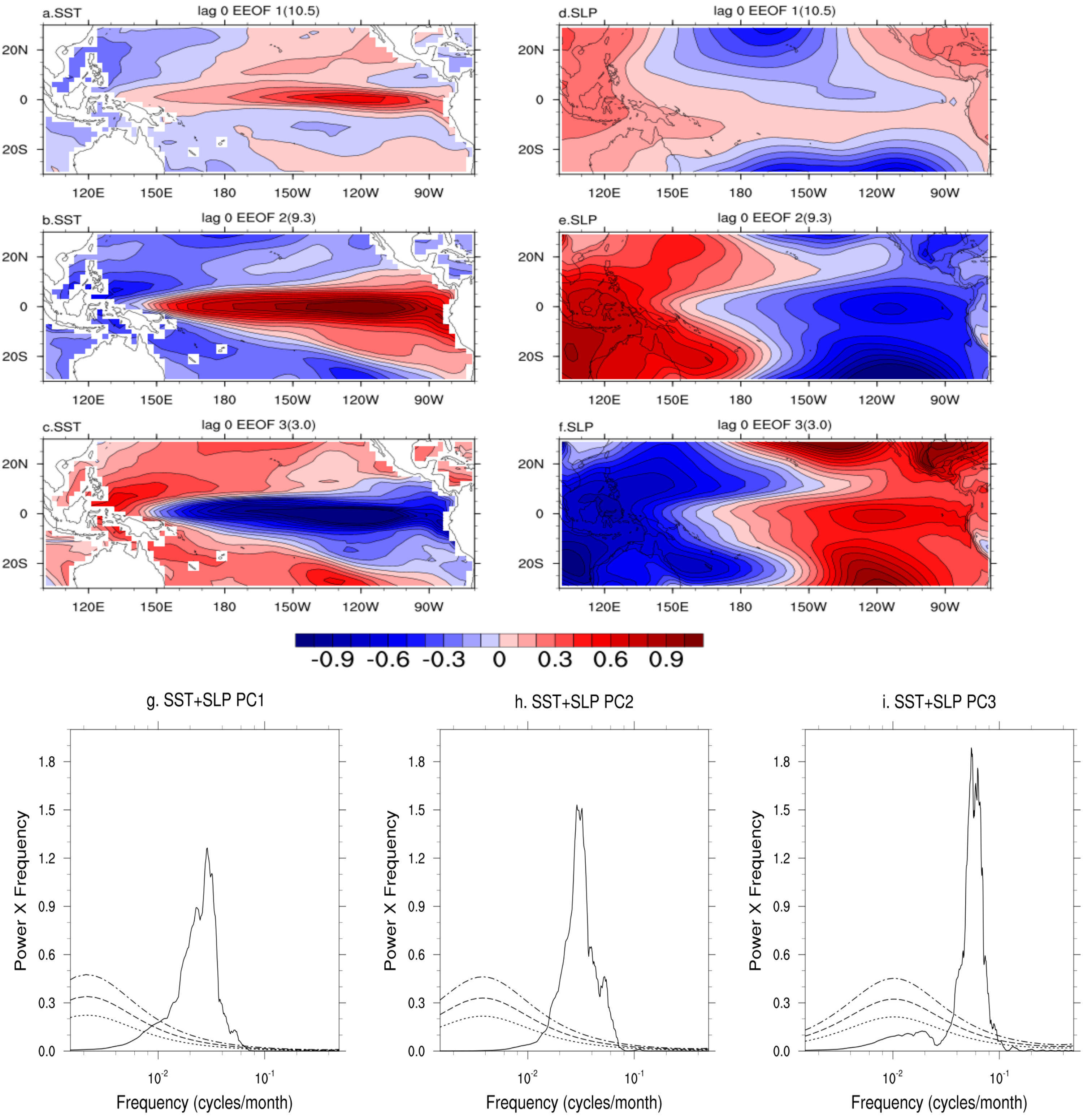

Figure S9: EEOFs and power spectra of PCs from GFDL long runs . (a)-(e)shows the EEOF patterns at lag 0 and (g)-(i) shows the power spectra of each of PC1,PC2 and PC3 respectively (cf.Fig.1). The combined EEOF analysis is based on monthly data of SST and SLP from 200 years of GFDL preindustrial control (PI) run with the 1850 greenhouse forcing as per as CMIP5 protocols. Monthly data from 200 years after the first hundred years have bemused for the EEOF analysis. Preprocessing and other steps for doing the EEOF analysis from the GFDL data are similar to what is done for EEOF analysis based on HADSLP and ERSST reanalysis (see text).

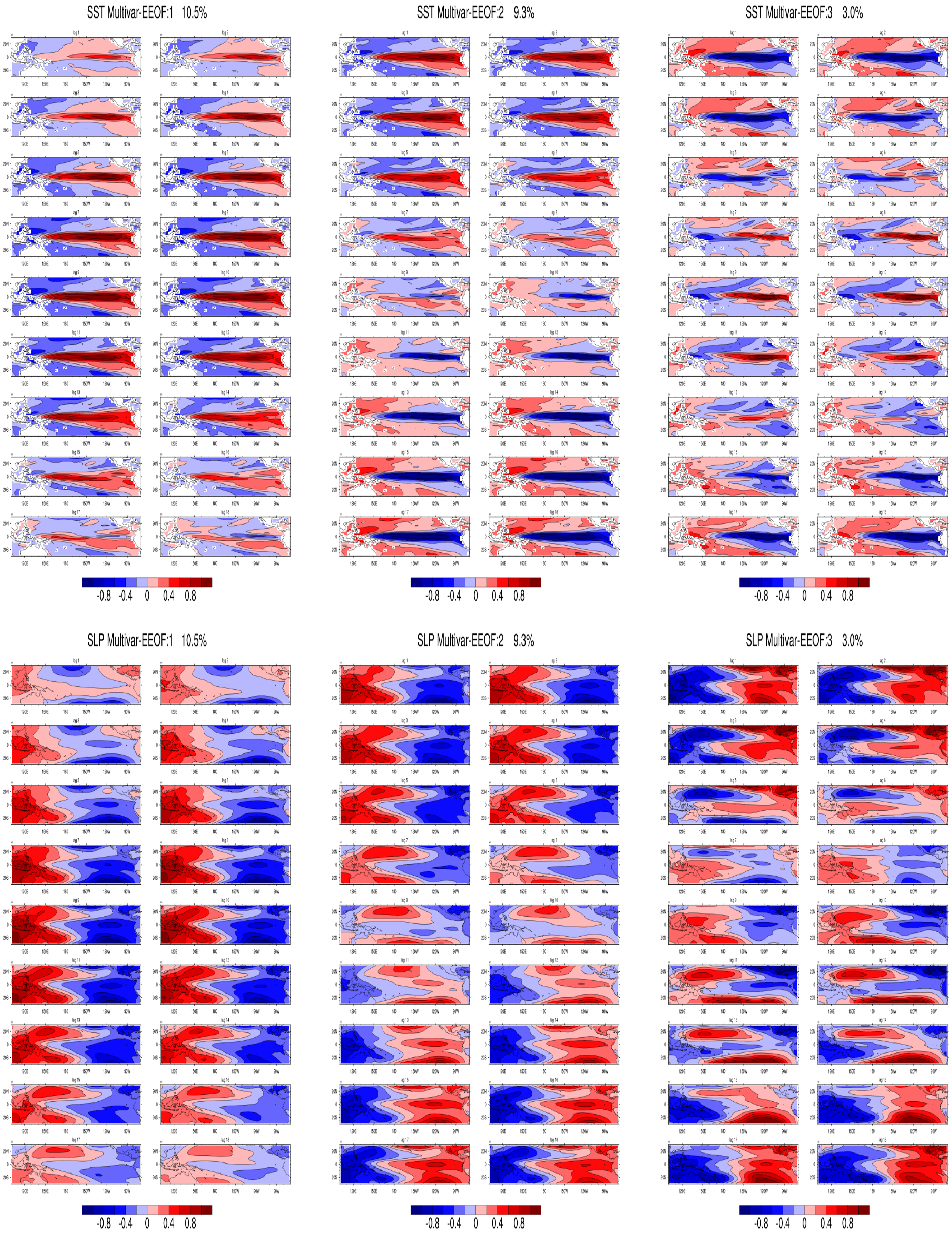

Figure S10: Evolutionary history of the three GFDL simulated EEOFs for all the 18 lags as considered in the last plot.

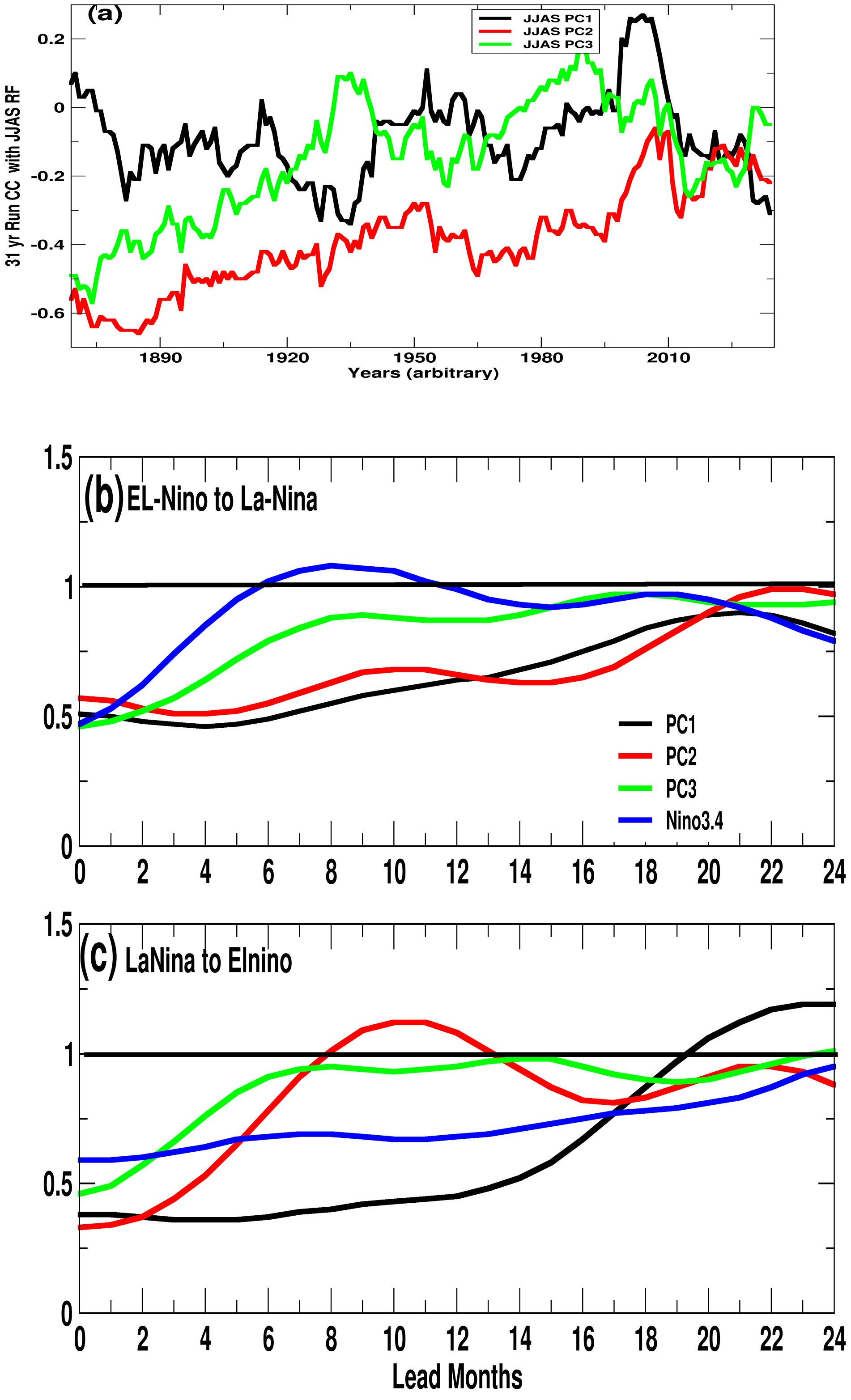

Figure S11: (a) 31 year running correlation between PC1, PC2 and PC3 and June-September averaged (JJAS) rainfall over the Indian region for the GFDL simulation.(b)Error growth from El Nino to La-Nina for GFDL simulated Nino3.4 and the three PCs from EEOF analysis of GFDL runs (cf. Fig. S12). (c)Same as (b) but for transitions from peak La Nina to El Nino. Fig.2(a) and Fig.4a-b may be referred for a comparison with observation/reanalysis.

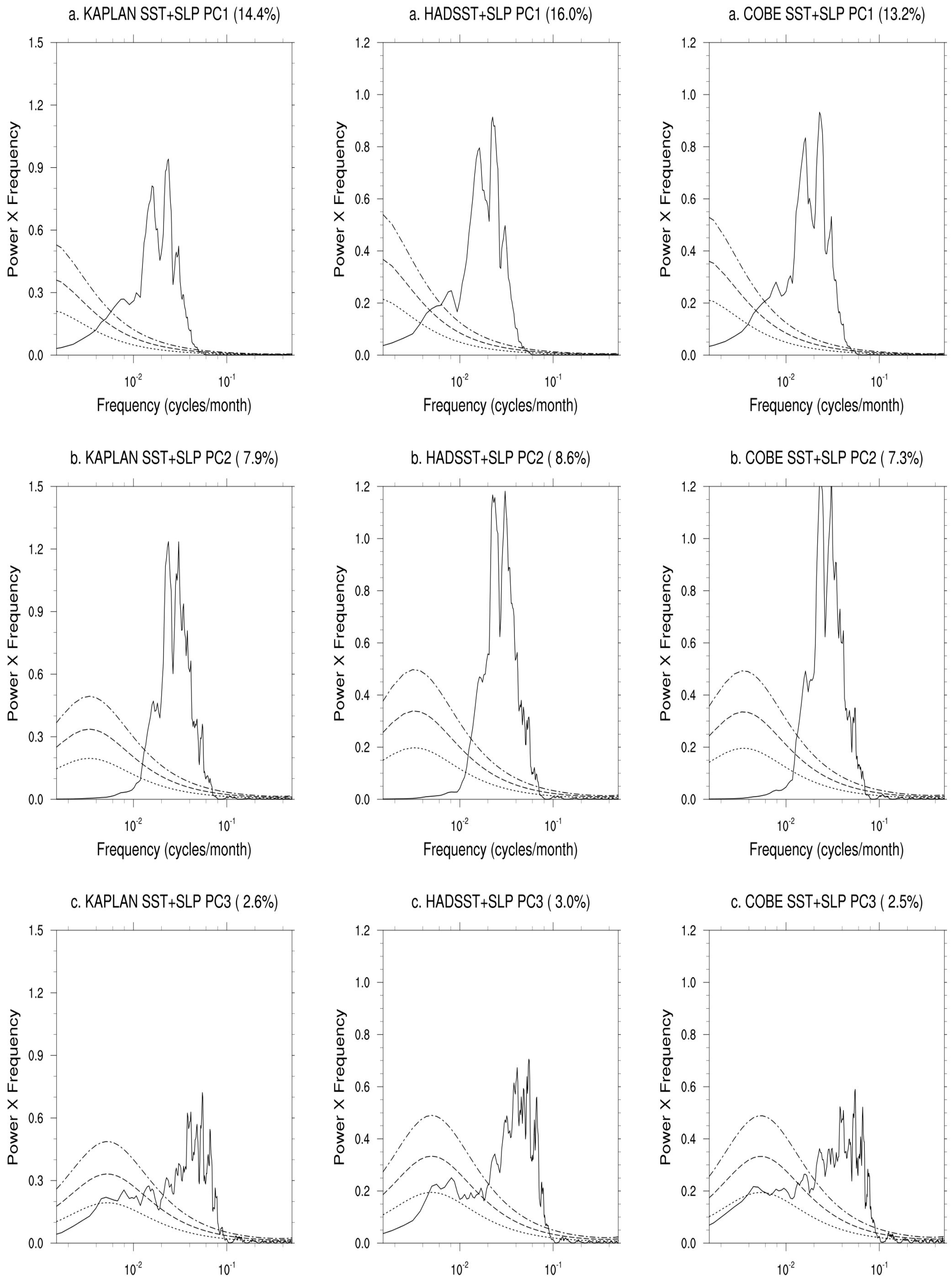

Figure S12: Power spectra of PCs computed from multiple SST dataset.Each SST dataset is mentioned at the top of each panel.

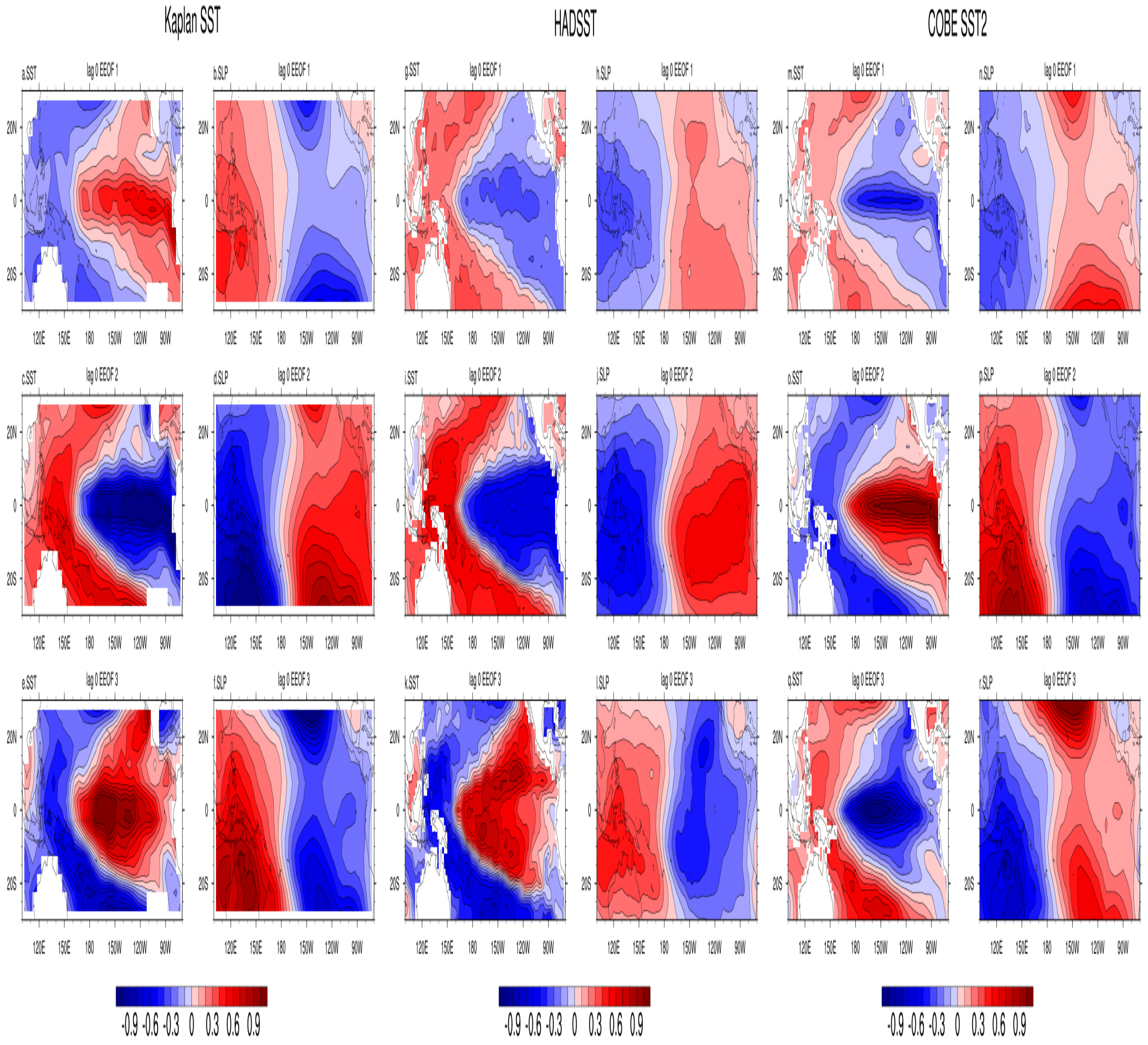

Figure S13: First three EEOFs (numbered top to bottom) of SST and SLP based Kaplan, HADLSST and COBE SST2.
